# Fruit, Vegetable, and Physical Activity Guideline Adherence and Metabolic Syndrome in El Banco por Salud

**DOI:** 10.3390/nu14091767

**Published:** 2022-04-23

**Authors:** Carrie S. Standage-Beier, Bahar Bakhshi, Oscar D. Parra, Lisa Soltani, Douglas J. Spegman, Patty Molina, Eladio Pereira, Lori Landes, Lawrence J. Mandarino, Lindsay N. Kohler

**Affiliations:** 1 School of Nutritional Sciences and Wellness, University of Arizona, Tucson, AZ 85721, USA; cstandagebeier@arizona.edu (C.S.S.-B.); baharbakhshi@arizona.edu (B.B.); 2 Center for Disparities in Diabetes, Obesity and Metabolism, University of Arizona, Tucson, AZ 85721, USA; oscardp@arizona.edu (O.D.P.); mandarino@email.arizona.edu (L.J.M.); 3 El Rio Community Health Centers, Tucson, AZ 85712, USA; lisas@elrio.org (L.S.); douglasjs@elrio.org (D.J.S.); 4 Mariposa Community Health Centers, Tucson, AZ 85621, USA; pmolina@mariposachc.net (P.M.); epereira@mariposachc.net (E.P.); 5 Department of Family Medicine and Population Health, Virginia Commonwealth University, Richmond, VA 23284, USA; lori.landes@vcuhealth.org; 6 Department of Epidemiology and Biostatistics, University of Arizona, Tucson, AZ 85721, USA; 7 Department of Health Promotion Sciences, University of Arizona, Tucson, AZ 85721, USA

**Keywords:** Hispanic/Latino, biobank, diet, physical activity, metabolic syndrome

## Abstract

Adherence to dietary and physical activity recommendations has been associated with reductions in morbidity and mortality. The association between baseline adherence to fruit, vegetable, and physical activity guidelines and metabolic syndrome (MetS) in El Banco por Salud (El Banco) was examined. El Banco is a wellness biobank for Latino individuals affiliated with partnered Federally Qualified Health Centers in southern Arizona. Study participants (*n* = 972) were 65% female, 62.3% foreign-born, 56.3% obese, 29.2% food insecure, and with an average age of 51.3 years. Adherence scores were developed using baseline questionnaires for fruits and vegetable consumption and self-reported physical activity. Adherence was low in those fully meeting guidelines for fruit, vegetable, and physical activity at 14.6%, 37.5%, and 23.5%, respectively. Roughly 65% (*n* = 630) had ≥3 cardiometabolic risk factors. Large waist circumference was the most prevalent risk factor at 77.9%. Adherence to physical activity recommendations differed by MetS status with 32.8% without MetS reporting ≥150 min of physical activity per week compared to 18.5% in those with MetS (*p* < 0.001). There were no significant associations with adherence to any guidelines and MetS in the fully adjusted model. Overall, in this sample guideline adherence was low and the cardiometabolic risk factors prevalence was high.

## 1. Introduction

Metabolic syndrome (MetS), a cluster of interrelated metabolic abnormalities, is associated with a 5-fold increase in risk of type 2 diabetes (T2D), and a 2-fold increase in risk of cardiovascular disease [1]. With increasing trends, MetS incidence often parallels that of T2D and obesity, but MetS prevalence is 3-times T2D prevalence [2]. According to the National Health and Nutrition Examination Survey (NHANES) data from 2011 to 2016, MetS prevalence was estimated at 34.5% in adults aged 20 and over [3]. Notably, across racial/ethnic groups, MetS had the second highest prevalence in Hispanics/Latinos (36.3%) [3]. Based on CDC data in 2018, 13.0% of American adults (18 and over) had diabetes, and Hispanic/Latinos had a disproportionately higher prevalence of T2D than non-Hispanic whites [1]. As a growing public health concern, MetS prevention and management relies upon lifestyle modifications, including long-term adherence to dietary and physical activity guidelines [4]. Fruit and vegetable intake comprise integral parts of healthful diets, and higher intakes have been observationally associated with reductions in risk of MetS [5] and T2D [6,7]. Likewise, moderate-to-vigorous physical activity (MVPA) has been inversely related to development of cardiometabolic risk factors [8]. 

To date, few studies have investigated (1) adherence to the fruit and vegetable recommendations based on Dietary Guidelines for Americans in Hispanic/Latino communities at high risk for T2D; (2) adherence to The US Department of Health and Human Services (USDHHS) Physical Activity guidelines in Hispanic/Latino communities at high risk for T2D; or (3) the association between adherence to fruit and vegetable and physical activity guidelines and MetS prevalence in Hispanic/Latino communities at high risk for T2D. In this study, we analyzed cross-sectional data to evaluate the association between adherence to fruit, vegetable, and physical activity recommendations and MetS among Latinos enrolled in the biobank, El Banco por Salud (El Banco). 

## 2. Materials and Methods 

### 2.1. Study Sample

El Banco is a biobank established by the University of Arizona Health Sciences Center for Disparities in Diabetes, Obesity, and Metabolism (CDDOM) in 2017 with ongoing recruitment. El Banco includes mostly patients that self-identify with Latino heritage (98%) from federally qualified health center (FQHC) partners in southern Arizona. Eligible patients are prescreened from their electronic health record for self-reported Latino ethnicity, age 18–75 years, and a HbA1c of 5.7 or greater. Recruited patients then served as probands in the study design. Family (including significantly close friends considered kin/family) were also recruited with no inclusion criteria for HbA1c. All participants provided written informed consent. Probands and family members are linked through a unique identifier for the family unit. Exclusion criteria includes (1) history of cancer, excluding non-melanoma skin cancers, in the past 3 years; (2) currently pregnant, delivered a baby within the last 12-months, or currently breastfeeding; and (3) feel they are unable to refrain from smoking for 1.5 h. Patients enrolled in El Banco provide samples of blood and saliva, complete an in-person comprehensive health-related questionnaire (modified Mayo Clinic Biobank Questionnaire) [2], and consent for access to medical records and recontact for future studies. Food security status was evaluated by the US Household Food Security Survey Module [3]. Enrollment is ongoing and as of November 2021 there were 990 patients enrolled. This study which examined fruit, vegetable, and physical activity guideline adherence and MetS (*n* = 972) was reviewed and approved by the University of Arizona Human Subjects Protection Program. The REDCap electronic data management system at the University of Arizona was used for data capture, quality assurance and control, as well as data export.

### 2.2. Dietary Assessment

A limited series of dietary questions are included in the baseline health history questionnaire. Average daily fruit and vegetable intake was determined through coding questionnaire responses for consumption rates of fruits and vegetables over an average week during the past month. Responses were coded into daily average fruit and vegetable intake by dividing the total weekly value by 7. Average daily values were categorized into categories using the Dietary Guidelines for Americans 2020–2025 values for daily consumption of total fruits and total vegetables. Participants fully met fruit guidelines if they consumed on average ≥2 servings per day over a 7-day week and met vegetable guidelines if they consumed on average ≥2.5 servings over a 7-day week. Partially meeting the fruit guidelines was defined as average consumption of 1–2 servings per day and not meeting was defined as averaging 0 servings per day. Few participants (*n* = 11) reported consuming no vegetables, therefore only two categories were defined for vegetables with those reporting an average 2.5 servings of vegetables considered as meeting recommendations

### 2.3. Physical Activity 

Physical activity was assessed through asking frequency of at least 15-min bouts of strenuous (rapid heartbeat), moderate, and mild level of exercise intensity over 7-days [4]. Questionnaire responses were coded into conservative number of total minutes by using the minimum 15 min for each bout of leisure time physical activity times the frequency reported in one week. Strenuous activity bouts were multiplied by two and added to participants’ moderate and mild minutes to assess the general 150 min/per week physical activity guideline. Participants were then categorized into fully meeting (≥150 min/week), partially meeting (15–135 min/week), and not meeting (0 min/week), using the USDHHS physical activity guidelines [5]. 

### 2.4. Cardiometabolic Risk Factors

MetS was defined according to the Adult Treatment Panel III [6], as having 3 or more of the following cardiometabolic risk factors; dyslipidemia (triglycerides ≥150 mg/dL), elevated fasting blood glucose (≥5.6 mmol/L), high-density lipoproteins (HDL) cholesterol (<40 mg/dL for men and <50 mg/dL for women), hypertension (systolic blood pressure > 130 mmHg and diastolic > 85 mmHg), large waist circumference (men ≥ 40 inch and women ≥ 35 inch). Each cardiometabolic risk factor was coded as either “1” for having factor or “0” for not having the factor. Participants that presented ≥3 cardiometabolic risk factors were deemed to have MetS. All measurements were performed by trained study staff in a clinical laboratory setting. 

### 2.5. Statistical Analysis

Descriptive statistics were generated for participant characteristics. Chi-square and t-tests were used to compare categorical and continuous variables by MetS status, respectively. Mixed effects logistic regression models were used to assess the relationship between adherence to fruit, vegetable, and physical activity guidelines and (1) MetS and (2) to evaluate potential interaction between adherence to each recommendation with sex and enrollment site. Mixed effect models included fixed effects for individual level covariates and a random family unit effect. Variables assessed for potential confounding were age, sex, education, site of enrollment, US born, general health status, insurance, marital status, sedentary time at work, and food security status. Sensitivity analyses were performed to exclude fruit juice and potatoes from the fruit and vegetable components, respectively. All analyses were performed with Status 16.1 (StataCorp, College Station, TX, USA). 

## 3. Results

Enrollment characteristics according to MetS status are presented in Table 1. Those with MetS (at least three cardiometabolic risk factors) were significantly older than those without MetS, more likely to have been enrolled at El Rio as a patient, be a proband, have worse cardiometabolic risk factors including higher BMI, poorer self-reported general health, have less than a high school education, have Medicare, have been born in the US, and be widowed or divorced. Sex, language spoken at home, work sedentary time, and food security status did not differ significantly by MetS status. 

### 3.1. Cardiometabolic Risk Factors in El Banco por Salud

**Figure 1:**
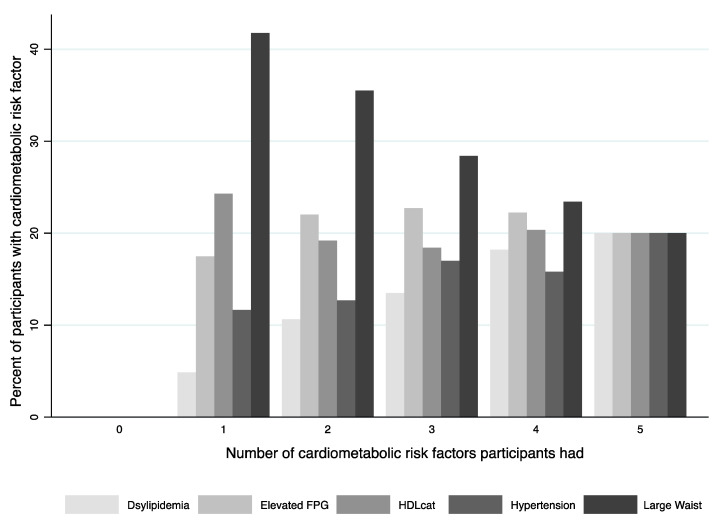
Prevalence of each cardiometabolic risk factor based on number of factors a participant presented.

The prevalence of obesity in El Banco is high with 56% of participants considered obese (BMI ≥ 30 kg/m^2^), 33% overweight (25 ≤ BMI < 30) and only 11% considered lean with a BMI < 25 kg/m^2^. The average BMI was 33.5 kg/m^2^ for those with three or more cardiometabolic risk factors (MetS) versus 29.5 kg/m^2^ for those with less than three risk factors (no MetS) (*p* < 0.001). Figure 1 shows the percentage of each cardiometabolic risk factor by the number of factors a participant presented. There were no participants with zero cardiometabolic risk factors. Large waist circumference was the most prevalent risk factor for participants with one to four risk factors while hypertension was the least prevalent. 

### 3.2. Dietary and Physical Activity Guidlies Adherence 

Adherence to fruit, vegetable, and physical activity guidelines was low in El Banco (Table 2). According to the self-report enrollment survey which included a limited series of dietary questions, overall, 14.6% were determined as fully meeting fruit consumption recommendations, 37.5% vegetable consumption recommendations and 23.5% physical activity guidelines. There was a statistically significant difference (*p* < 0.001) in adherence to physical activity guidelines by MetS status with 32.8% of the participants with no MetS meeting the recommendation of ≥150 min/week compared to those with MetS (18.5%). 

The unadjusted and adjusted odds ratios for the association between adherence to fruit, vegetable, and physical activity recommendation and MetS are presented in Table 3. Model 1 is mutually adjusted for adherence to recommendations and demonstrates a statistically significant reduction in odds of MetS in those meeting the recommendations for physical activity (OR = 0.43; 95% CI: 0.29–0.64; *p*-trend < 0.001). Model 2 is further adjusted for age, sex, insurance status, self-reported general health, US born, high school education or less, and enrollment site. Estimates for meeting physical activity recommendations were attenuated (OR = 0.69; 95% CI: 0.45–1.05) and no longer reached statistical significance (*p* = 0.086). The association between adherence to fruit and vegetable consumption and MetS did not meet statistical significance in either model or tests for trend. No significant interactions were observed by sex or enrollment site. 

### 3.3. Sensitivity Analyses

Excluding fruit juice from fruit consumption and potatoes from vegetable consumption statistically significantly changed adherence to recommendations (*p* < 0.001). After exclusion of fruit juice, over 33% of individuals who already fully met the fruit recommendations, and over 7% of those who partially met the recommendations dropped out of their initial adherence categories. Similarly, excluding potatoes resulted in 47% drop out of individuals who already fully met vegetable recommendations. No important differences were seen in logistic regression estimates when fruit juice and potatoes were excluded from fruit and vegetable intake for the fully adjusted association between adherence to fruit, vegetable, or physical activity guidelines and MetS.

## 4. Discussion

The study results demonstrate that adherence to recommendations for fruit and vegetable intake as well as physical activity is low in El Banco por Salud. Participants reporting ≥150 min per week of leisure time physical activity (meeting guidelines) had a statistically significant reduction in odds of MetS compared to participants reporting no leisure time physical activity (not meeting guidelines) in a mutually adjusted model with adherence to fruit and vegetable recommendations. This is most likely a conservative estimate with the assumption of each bout of exercise lasting the minimum of only 15 min. However, after adjusting for confounding factors the effect was attenuated and did not reach statistical significance. There were no significant associations demonstrated for guideline adherence for fruit or vegetable consumption and MetS in the mutually adjusted and fully adjusted models.

To our knowledge, no studies have evaluated the association between adherence to fruit, vegetable, and physical activity guidelines and MetS in Latino communities. However, prior studies have shown an inverse association of adherence to dietary and physical activity guidelines with MetS. Recently, the Framingham Heart Study, a largely non-Hispanic, white sample, suggested a synergistic impact of diet, indicated as Dietary Guidelines for American Adherence Index (DGAI), and physical activity on cardiometabolic health, showing that higher adherence to both DGAI and physical activity guidelines is associated with greater reduction in the odds of MetS [7]. Based on a systematic review of observational studies [8], objectively measured MVPA is inversely associated with MetS. However, literature examining this association in Hispanic/Latino populations has been inconsistent. A study of Hispanic/Latino adults observed a non-statistically significant increase in MetS components with lower levels of MVPA [9]. Similarly, among Hispanic/Latinos participating in the Dallas Heart Study, MVPA was negatively associated with metabolic risk factors, although for a number of MetS components, the reduction in trends did not reach statistical significance [10].

Guidelines defining MetS within this literature included the American Heart Association/National Heart, Lung, and Blood Institute guidelines [7,8], the Harmonized guidelines [11] and the Adult Treatment Panel III [8]. Differences in definitions of MetS were that the American Heart Association/National Heart, Lung, and Blood Institute and the Harmonized guidelines allowed for non-anthropometric risk factors to be determined by either laboratory values or specific medication use [7,8]. Harmonized guidelines also contained population and country specific definitions for elevated waist circumference criteria [8]. While the Adult Treatment Panel III was used to define MetS based on standardized laboratory values/anthropometric measures [6].

According to an analysis of the 2015 Behavioral Risk Factor Surveillance System (BRFSS), the percentage of Hispanic/Latinos who met the federal fruit and vegetable recommendations corresponded to 15.7% and 10.5%, respectively [12]. In this study, a higher proportion of participants fully adhered to the recommendations; however, the overall adherence to the fruit and vegetable guidelines remained relatively low. Fruit and vegetable consumption, from a single-nutrient perspective, has been linked to reduced odds of MetS in systematic reviews and meta-analysis of observational studies [13,14,15]. Highest vs. lowest consumption of fruit, vegetable, and combined fruit and vegetable were associated with 11%, 19%, and 25% reduction in risk of MetS, respectively [15]. Additionally, fruit consumption has been linked to MetS risk in a dose–response manner, where a 100 g/d increase in fruit intake (approximately one serving equivalent) reduced the risk of MetS by 3% [13]. In the present study, lack of association between adhering guidelines and odds of MetS may be due to the limited series of self-reported dietary and physical activity questions asked during the biobank enrollment.

The major strengths of the current study include a large sample size of Latino participants enrolled in El Banco por Salud, a biobank specifically designed to study cardiometabolic health in Latino individuals, substantial self-reported health data as well as clinical and anthropometric measurements, and the ability to recontact these participants in the future. Moreover, the HEI-2015 is a valid and reliable metric to measure diet quality based on adherence to national dietary guidelines, including fruit and vegetable recommendations, among ethnic groups, such as Hispanic/Latinos [16]. However, this study is not without limitations. The cross-sectional nature of the study cannot determine temporality between MetS and adherence to guidelines since the data were collected at the same time for enrollment in the biobank. Self-reported measurements of physical activity may be associated with recall bias, and over- or underestimation of physical activity time and/or intensity. Additionally, self-reported fruit and vegetable intake may cause underestimation of the MetS odds [17]. Smoking status was evaluated as either ever or never but did not impact the estimates and therefore was not included in the final model. However, it is known to be a risk factor for metabolic syndrome [18]. Alcohol consumption may also play a role in MetS but alcohol consumption in El Banco was low with participants reporting consuming a drink with alcohol once per month or less (27.7%) or no consumption at all (77.2%) in the past 12 months. Further, there were limited diet and supplementation questions included in the enrollment questionnaire. Serving size was not collected and diet questions were not linked to a nutrient database, thus overall energy intake could not be assessed. Due to the nature of the biobank, participants could be recalled and at a minimum, a subsample should be assessed more thoroughly for dietary intake using 24-hour dietary recalls. The implementation of 24-hour recalls would be linked to a nutrient database allowing for full computation of the HEI-2015.

## 5. Conclusions

In summary, results suggest that fruit and vegetable intake and leisure time physical activity in El Banco are below recommendations for a healthy lifestyle in a population with or at-risk for MetS. Further research is needed to better determine the association between fruit, vegetable, and physical activity guideline adherence and MetS in Latino communities.

## Figures and Tables

**Table 1:** El Banco por Salud participant characteristics by metabolic syndrome status (*n* = 972).

	Overall *	No MetS	MetS	*p*-Value
	*n* = 972	*n* = 342	*n* = 630	
Age, years	51.3 (15.1)	45.4 (16.4)	54.5 (13.2)	<0.001
Enrollment Site				<0.001
El Rio	729 (75.0%)	233 (68.1%)	496 (78.7%)	
Mariposa	243 (5.0%)	109 (31.9%)	134 (21.3%)	
Participant type				<0.001
Proband	455 (46.8%)	98 (28.7%)	357 (56.7%)	
Family Member	517 (53.2%)	244 (71.3%)	273 (43.3%)	
Sex				0.663
Male	321 (33.0)	116 (33.9%)	205 (32.5%)	
Female	651 (67.0)	226 (66.1%)	425 (67.5%)	
Cardiometabolic Risk Factors				
Body mass index (kg/m^2^)	32.1 (6.8)	29.5 (6.4)	33.5 (6.6)	<0.001
Waist circumference (inches)	40.8 (6.2)	37.4 (5.7)	42.9 (5.7)	<0.001
Fasting plasma glucose (mmol/L)	8.3 (4.3)	6.3 (3.0)	9.3 (4.6)	<0.001
HDL cholesterol (mg/dL)	46 (12)	52 (12)	42 (10)	<0.001
Triglycerides (mg/dL)	180 (174)	115 (59)	216 (203)	<0.001
Systolic blood pressure (mmHg)	132 (21)	120 (15)	138 (20)	<0.001
Diastolic blood pressure (mmHg)	79 (8)	75 (7)	81 (8)	<0.001
Insurance Type				<0.001
Medicaid	427 (44.1%)	154 (45.2%)	273 (43.5%)	
Commercial	125 (12.9%)	40 (11.7%)	85 (13.5%)	
Medicare	229 (23.6%)	56 (16.4%)	173 (27.5%)	
None	83 (8.6%)	35 (10.3%)	48 (7.6%)	
Unknown	105 (10.8%)	56 (16.4%)	49 (7.8%)	
Marital status				0.006
Single, never married	213 (21.4%)	94 (27.6%)	117 (18.6%)	
Married or domestic partnership	528 (53.1%)	174 (51.0%)	342 (54.3%)	
Widowed	77 (7.7%)	16 (4.7%)	57 (9.0%)	
Divorced	126 (12.7)	40 (11.7%)	82 (13.0%)	
Separated	50 (5.0%)	17 (5.0%)	32 (5.1%)	
Education				<0.001
High school or less	756 (76.2%)	236 (30.6%)	501 (20.3%)	
Greater than high school	236 (23.8%)	104 (69.4%)	128 (79.7%)	
United States born	366 (37.9%)	144 (42.4%)	222 (35.5%)	0.035
Language spoken at home				0.784
Only Spanish	324 (33.5%)	105 (31.0%)	219 (34.9%)	
More Spanish than English	259 (26.8%)	93 (27.4%)	166 (26.4%)	
Both equally	202 (20.9%)	76 (22.4%)	126 (20.1%)	
More English than Spanish	122 (12.6%)	43 (12.7%)	79 (12.6%)	
Only English	60 (6.2%)	22 (6.5%)	38 (6.1%)	
Work sedentary (sitting) time				0.393
All of the time	55 (5.7%)	13 (3.8%)	42 (6.7%)	
Most of the time	151 (15.6%)	57 (16.7%)	94 (15.0%)	
Some of the time	254 (26.2%)	91 (26.6%)	163 (26.0%)	
A little of the time	263 (27.2%)	97 (28.4%)	166 (26.5%)	
None of the time	245 (25.3%)	84 (24.6%)	161 (25.7%)	
Food Insecurity	274 (29.2%)	97 (29.5%)	177 (29.0%)	0.8806
Self-reported general health				
Excellent	51 (5.3%)	25 (7.3%)	26 (4.1%)	<0.001
Very good	119 (12.3%)	69 (20.2%)	50 (8.0%)	
Good	332 (34.3%)	129 (37.8%)	203 (32.3%)	
Fair	310 (32.0%)	86 (25.2%)	224 (35.7%)	
Poor	157 (16.2%)	32 (9.4%)	125 (19.9%)	

* Means (standard deviations) or frequency (%).

**Table 2:** Adherence to dietary and physical activity guidelines by metabolic syndrome status in El Banco por Salud.

	No MetS (*n* = 342)	MetS (*n* = 630)	Overall (*n* = 972)	*p*-Value
Fruit Consumption				0.183
Not Meeting (0 servings/day)	26 (7.7%)	71 (11.4%)	97 (10.1%)	
Partially (1–2 servings/day)	264 (77.7%)	463 (74.1%)	727 (75.%)	
Meeting (≥2 servings/day)	50 (14.7%)	91 (14.6%)	141 (14.6%)	
Vegetable Consumption				0.176
Not Meeting (<2.5 servings/day)	219 (65.4%)	379 (60.9%)	598 (32.5%)	
Meeting (≥2.5 servings/day)	116 (34.6%)	243 (39.1%)	359 (37.5%)	
Physical Activity				<0.001
Not Meeting (0 min/week)	70 (20.5%)	161 (25.8%)	231 (23.9%)	
Partially (15–135 min/week)	160 (46.8%)	347 (55.7%)	507 (52.4%)	
Meeting (≥150 min/week)	112 (32.8%)	115 (18.5%)	227 (23.5%)	

**Table 3:** Odds of metabolic syndrome by adherence to fruit, vegetable, and physical activity recommendations.

	Model 1 ^a^		Model 2 ^b^			
	OR (95% CI)		*p*-Value	OR (95% CI)		*p*-Value
Fruit Consumption						
Not Meeting (0 servings/day)	Reference			Reference		
Partially (1–2 servings/day)	0.61	(0.38–0.99)	0.045	0.70	(0.42–1.17)	0.174
Meeting (≥2 servings/day)	0.60	(0.33–1.07)	0.085	0.75	(0.40–1.40)	0.363
*p*-trend ^c^			0.163			0.518
Vegetable Consumption						
Not Meeting/Partially (<2.5 servings/day)	Reference			Reference		
Meeting (≥2.5 servings/day)	1.23	(0.93–1.65)	0.149	1.05	(0.77–1.44)	0.751
Physical Activity						
Not Meeting (<15 min/week)	Reference			Reference		
Partially (15–135 min/weeek)	0.96	(0.68–1.35)	0.810	1.02	(0.71–1.48)	0.905
Meeting (≥150 min/week)	0.43	(0.29–0.64)	<0.001	0.69	(0.45–1.05)	0.086
*p*-trend			<0.001			0.102

^a^ Mutually adjusted for recommendations. ^b^ Model 1 additionally adjusted for age, sex, insurance, self-reported general health, US born, education, enrollment site. ^c^ *p*-trend calculated using regression modeling with recommendations as a continuous variable.

## Data Availability

The data captured for this biobank can be made available by the authors with approval from the CDDOM Biobank Executive Joint Governance Committee. For details please visit: https://cddom.uahs.arizona.edu/banco-por-salud-wellness-bank/requesting-biobank-data-and-samples.

## References

[1] U.S. Department of Health and Human Services (2020). National Diabetes Statistics Report.

[2] Olson J.E., Ryu E., Johnson K.J., Koenig B.A., Maschke K.J., Morrisette J.A., Liebow M., Takahashi P.Y., Fredericksen Z.S., Sharma R.G. (2013). The Mayo Clinic Biobank: A building block for individualized medicine. Mayo Clin. Proc..

[3] U.S. Adult Food Security Survey Module: Three-Stage Design, with Screeners: USDA. https://www.ers.usda.gov/media/8279/ad2012.pdf.

[4] Godin G., Shephard R.J. (1985). A simple method to assess exercise behavior in the community. Can. J. Appl. Sport Sci..

[5] Piercy K.L., Troiano R.P., Ballard R.M., Carlson S.A., Fulton J.E., Galuska D.A., George S.M., Olson R.D. (2018). The Physical Activity Guidelines for Americans. JAMA.

[6] Grundy S.M., Cleeman J.I., Daniels S.R., Donato K.A., Eckel R.H., Franklin B.A., Gordon D.J., Krauss R.M., Savage P.J., Smith S.C. (2005). Diagnosis and management of the metabolic syndrome: An American Heart Association/National Heart, Lung, and Blood Institute Scientific Statement. Circulation.

[7] Lee J., Walker M.E., Bourdillon M.T., Spartano N.L., Rogers G.T., Jacques P.F., Vasan R.S., Xanthakis V. (2021). Conjoint associations of adherence to physical activity and dietary guidelines with cardiometabolic health: The Framingham Heart Study. J. Am. Heart Assoc..

[8] Amirfaiz S., Shahril M.R. (2019). Objectively measured physical activity, sedentary behavior, and metabolic syndrome in adults: Systematic review of observational evidence. Metab. Syndr. Relat. Disord..

[9] Mossavar-Rahmani Y., Hua S., Qi Q., Strizich G., Sotres-Alvarez D., Talavera G.A., Evenson K.R., Gellman M.D., Stoutenberg M., Castañeda S.F. (2020). Are sedentary behavior and physical activity independently associated with cardiometabolic benefits? The Hispanic Community Health Study/Study of Latinos. BMC Public Health.

[10] Lakoski S.G., Kozlitina J. (2014). Ethnic differences in physical activity and metabolic risk: The Dallas Heart Study. Med. Sci. Sports Exerc..

[11] Alberti K.G.M.M., Eckel R.H., Grundy S.M., Zimmet P.Z., Cleeman J.I., Donato K.A., Fruchart J.-C., James W.P.T., Loria C.M., Smith S.C. (2009). Harmonizing the metabolic syndrome: A joint interim statement of the International Diabetes Federation Task Force on Epidemiology and Prevention; National Heart, Lung, and Blood Institute; American Heart Association; World Heart Federation; International Atherosclerosis Society; and International Association for the Study of Obesity. Circulation.

[12] Lee-Kwan S.H., Moore L.V., Blanck H.M., Harris D.M., Galuska D. (2017). Disparities in State-Specific Adult Fruit and Vegetable Consumption—United States, 2015. MMWR Morb. Mortal. Wkly. Rep..

[13] Lee M., Lim M., Kim J. (2019). Fruit and vegetable consumption and the metabolic syndrome: A systematic review and dose-response meta-analysis. Br. J Nutr..

[14] Tian Y., Su L., Wang J., Duan X., Jiang X. (2018). Fruit and vegetable consumption and risk of the metabolic syndrome: A meta-analysis. Public Health Nutr..

[15] Zhang Y., Zhang D.Z. (2018). Associations of vegetable and fruit consumption with metabolic syndrome. A meta-analysis of observational studies. Public Health Nutr..

[16] Jacobs S., Boushey C.J., Franke A.A., Shvetsov Y.B., Monroe K.R., Haiman C.A., Kolonel L.N., Le Marchand L., Maskarinec G. (2017). A priori-defined diet quality indices, biomarkers and risk for type 2 diabetes in five ethnic groups: The Multiethnic Cohort. Br. J. Nutr..

[17] Bothwell E.K., Ayala G.X., Conway T.L., Rock C.L., Gallo L.C., Elder J.P. (2009). Underreporting of food intake among Mexican/Mexican-American Women: Rates and correlates. J. Am. Diet Assoc..

[18] Cena H., Fonte M.L., Turconi G. (2011). Relationship between smoking and metabolic syndrome. Nutr. Rev..

